# Methodologies toward Efficient and Stable Cesium Lead Halide Perovskite‐Based Solar Cells

**DOI:** 10.1002/advs.201800509

**Published:** 2018-06-20

**Authors:** Jae Keun Nam, Do Hyung Chun, Ryan Joon Kyu Rhee, Jung Hwan Lee, Jong Hyeok Park

**Affiliations:** ^1^ Department of Chemical and Biomolecular Engineering Yonsei University Seoul 03722 Republic of Korea

**Keywords:** cesium lead halide perovskites, phase stability, photophysics, solar cells, thin film fabrication

## Abstract

In an attempt to replace thermally vulnerable organic perovskites, considerable research effort has recently been focused on the development of all‐inorganic perovskites in the field of photovoltaics. The preceding studies demonstrated that cesium lead halide perovskites are promising candidates for thermally stable and efficient solar cell materials. Here, the recent progress in cesium lead halide perovskite‐based solar cells is summarized. Whether organic cations are essential for the superiority of halide perovskites is controversial. However, more than 13% efficient solar cells have been successfully fabricated by employing cesium lead halide perovskites in a short amount of time. The state‐of‐the‐art materials engineering techniques will help to achieve a remarkable photovoltaic performance comparable to that of organic perovskites. In addition, improved understanding of the intrinsic photophysical behaviors will provide new insights that will facilitate further improvements in solar cell applications.

## 1 Introduction

Halide perovskites are of great interest in the photovoltaic research field. Many research efforts have brought a sharp growth, achieving superior power conversion efficiency (PCE) above 22%.^1^, ^2^, ^3^, ^4^, ^5^, ^6^, ^7^, ^8^, ^9^, ^10^ The general formula of this material is ABX_3_, where A is a monovalent cation, B is typically Pb, and X is a halide.^11^ As a prototypical material, organic cation‐based methylammonium (MA) lead iodide (CH_3_NH_3_PbI_3_, denoted MAPbI_3_) possesses optoelectronic properties that are essential for solar cells. This material shows strong optical absorption (bandgap of 1.5 eV) and ambipolar characteristics (light electron and carrier masses).^12^, ^13^ In addition, the large dielectric constant attributes to charge carrier separation (weak exciton) and transport (low scattering).^14^, ^15^ Compositional modifications such as mixed‐cation, mixed‐halide, and nonstoichiometric approach can build state‐of‐the‐art halide perovskite films. For example, the incorporation of formamidinium (FA) or inorganic cations (Cs or Rb) into bare MAPbI_3_ (e.g., FA_0.9_Cs_0.1_PbI_3_, (FAPbI_3_)_0.85_(MAPbBr_3_)_0.15_) gives an exceptional photovoltaic performance and structural stability.^9^, ^16^, ^17^ The development of deposition techniques, such as two‐step sequential method, co‐evaporation, and antisolvent‐washing, facilitates the rapid growth of perovskite photovoltaics, by forming pinhole‐free, large‐grained polycrystalline halide perovskite thin films.^4^, ^5^, ^6^

In spite of the superior efficiency of organic perovskites, stability issues still remain. For instance, the CH_3_NH_3_PbI_3_ film degrades at temperature above 85 °C.^18^, ^19^ This point is a critical flaw for photovoltaic application, which requires stability over a wide range of operating temperatures. Various approaches have been developed to form thermally stable perovskites. For example, the incorporation of FA or inorganic cations like Cs or Rb improves the thermal stability of MA‐based perovskites.^9^, ^16^, ^17^ However, the volatility of organic molecules may be an ultimate hurdle for commercialization. Concerns about this stability issue have stimulated research motivation for all‐inorganic perovskites. Since 2015, high‐performance cesium lead halide (CLH) perovskite‐based solar cells have begun to be reported.^20^, ^21^, ^22^, ^23^, ^24^, ^25^, ^26^, ^27^, ^28^, ^29^ Tailored composition and additive studies have led to a high photovoltaic efficiency as well as improved structural stability. The application of various deposition techniques enables the fabrication of high‐quality CLH perovskite thin films. Moreover, photophysical analysis identifies the intrinsic differences between organic‐ and inorganic CLH perovskites. Taking all these reports into account, CLH perovskites clearly have numerous potential as thermally stable and efficient photovoltaic light absorbers.

In this review, the recent progress in CLH perovskite‐based solar cells is summarized. This review is divided into four parts: In Section 2, the compositionally modified CLH perovskites, including mixed‐halide, mixed‐cation, and non‐stoichiometric approach, are discussed. In Section 3, brief descriptions of solution‐, vacuum‐processed, and quantum dot (QD)‐based deposition techniques are given. In Section 4, photophysical properties of CLH perovskites, including their distinctive recombination and photoinduced ion migration kinetics, are discussed. Finally, our concluding remark and perspectives are given in Section 5.

## 2 Compositional Modification

The compositional modification of halide perovskites has led to significant improvements in two major aspects: From the photophysical point of view, the mixed‐cation and mixed‐halide approaches create desirable optical absorption, which is determined by the bandgap of halide perovskites. In terms of the crystal structure, the relative ionic size of cations and halides affects the structural stability of ABX_3_ perovskite lattice, which is represented by a tolerance factor.^11^ For CLH perovskites, both of these intrinsic characteristics have been major obstacles for driving further research since these have an undesirably large bandgap and notorious phase instability.^21^ In this section, the effectiveness of compositional modification on CLH perovskites is discussed, with respect to the photovoltaic performance and structural stability.

**Figure 1:**
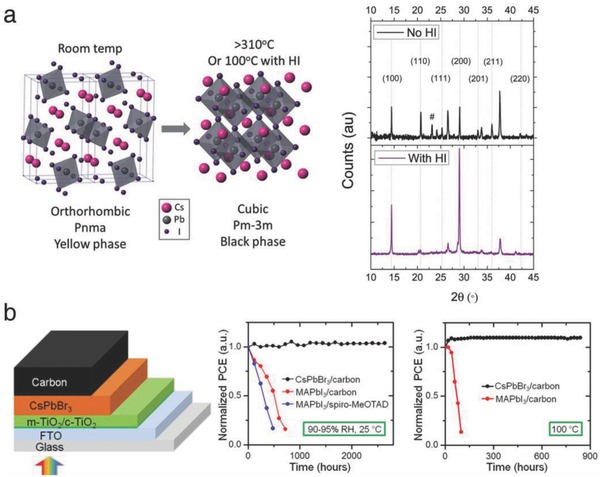
a) Schematic of the phase transition from the δ‐phase to the α‐phase, and XRD spectra of the CsPbI_3_ films with and without HI additive. Reproduced with permission.^20^ Copyright 2015, Royal Society of Chemistry. b) Device structure of an all‐inorganic–based perovskite solar cell, and comparison of the normalized PCEs of the CsPbI_3_‐ and CH_3_NH_3_PbI_3_‐based solar cells. Reproduced with permission.^39^ Copyright 2016, American Chemical Society.

**Table** **1** displays the previously reported CLH perovskite solar cells, sorted by the composition and deposition method. The early stage of research on all‐inorganic perovskite‐based solar cells employed either CsPbI_3_ or CsPbBr_3_. In 2015, Eperon et al. reported a working 2.9%efficient CsPbI_3_‐based solar cells for the first time.^20^ A small amount of HI in the precursor solution helped stabilize the perovskite black phase (α‐phase) with a more pronounced crystal orientation (**Figure** **1**a). On the basis of this finding, they suggested that CLH perovskites also possess weak excitons and sufficiently long charge carrier diffusion lengths. It was also speculated that the ferroelectricity of perovskites is not the reason for hysteresis behavior of perovskite‐based solar cells since the space group of CsPbI_3_ does not exhibit a ferroelectric property. At a similar time, Kulbak et al. demonstrated the CsPbBr_3_‐based solar cells with a high open‐circuit voltage (*V*_OC_) of 1.3 V.^38^ This group questioned whether the anisotropic geometry of MA is essential for the photovoltaic superiority of halide perovskites and suggested that fundamental study should be performed to unravel the comparative relationship between organic‐ and inorganic‐cation–based halide perovskites. Later, Liang et al. fabricated all‐inorganic–based solar cells employing the CsPbBr_3_/carbon system (Figure 1b).^39^ Without organic‐based charge transport materials, the solar cell reached a high PCE of 6.7% and showed an extremely long operating lifetime in the presence of heat and moisture (100 °C, 95% RH). Recently, similar approach was applied to simplified solar cells and bifunctional energy harvesting devices, exhibiting a comparable efficiency as well as superior ambient stability.^41^, ^49^

**Table 1:** Summary of the reported CLH perovskite‐based solar cells

Entry	Composition	Modification	Deposition method	Cell configuration	*J*_SC_ [mA cm^‐2^]	*V*_OC_ [V]	FF	PCE [%]
1^20^	CsPbI_3_		Spin‐coating	Au/spiro‐OMeTAD/perov/c‐TiO_2_/FTO	12.0	0.80	–	2.9
2^30^		+BiI_3_		Au/CuI/perov/c‐TiO_2_/FTO	18.8	0.97	0.73	13.2
3^31^		+CaI_2_		Au/P3HT/perov/mp‐,c‐TiO_2_/FTO	17.9	0.95	0.80	13.5
4^32^		+PEAI		Al/PCBM/perov/PEDOT:PSS/ITO	15.0	1.06	0.41	6.5
5^33^		+EDAPbI_4_		Ag/spiro‐OMeTAD/perov/c‐TiO_2_/FTO	14.5	1.15	0.71	11.9
6^34^		+zwitterions		Al/bcp/C_60_/PCBM/perov/PTAA/ITO	14.9	1.08	0.70	11.4
7^26^			Colloidal solution	Al/MoO*_x_*/spiro‐OMeTAD/perov/c‐TiO_2_/FTO	13.5	1.23	0.65	10.8
8^35^		+FAI		Al/MoO*_x_*/spiro‐OMeTAD/perov/c‐TiO_2_/FTO	14.4	1.20	0.78	13.4
9^36^			Vacuum evaporation	Ag/SWCNT/perov/SnO_2_/FTO	13.0	1.00	0.68	8.8
10^37^				Au/P3HT/perov/c‐TiO_2_/ITO	13.8	1.06	0.72	10.5
11^38^	CsPbBr_3_		Spin‐coating	Au/spiro‐OMeTAD/perov/mp‐TiO_2_/FTO	6.2	1.28	0.74	6.0
12^39^				Carbon/perov/mp‐, c‐TiO_2_/FTO	7.4	1.24	0.73	6.7
13^40^		+CsBr		Carbon/perov/GQDs/mp‐, c‐TiO_2_/FTO	8.1	1.46	0.82	9.7
14^25^			Colloidal solution	Au/spiro‐OMeTAD/perov/c‐TiO_2_/FTO	7.1	1.42	0.53	5.5
15^41^				Carbon/perov/GQDs/FTO	5.1	1.21	0.67	4.1
16^42^		+NH_4_SCN		Au/spiro‐OMeTAD/perov/ZnO/FTO	6.2	1.43	0.77	6.8
17^21^	CsPbI_2_Br		Spin‐coating	Ag/spiro‐OMeTAD/perov/c‐TiO_2_/FTO	11.9	1.11	0.75	9.8
18^22^				Al/bcp/PCBM/perov/PEDOT:PSS/ITO	10.9	1.12	–	6.7
19^24^				Au/spiro‐OMeTAD/perov/c‐TiO_2_/FTO	12.0	1.23	0.73	10.7
20^29^				Ag/ZnO‐C_60_/perov/NiO*_x_*/FTO	15.2	1.14	0.77	13.3
21^23^		+KI		Au/spiro‐OMeTAD/perov/c‐TiO_2_/FTO	11.6	1.18	0.73	10.0
22^43^		+SrI_2_		Au/P3HT/perov/mp‐,c‐TiO_2_/FTO	15.3	1.04	0.70	11.2
23^44^		+MnCl_2_		Carbon/perov/mp‐,c‐TiO_2_/FTO	14.4	1.17	0.80	13.5
24^45^			Colloidal solution	Au/PTAA/perov/c‐TiO_2_/FTO	12.9	1.19	0.81	12.4
25^27^			Vacuum evaporation	Ag/TAPC/MoO_3_/perov/C_60_/Ca/ITO	15.2	1.15	0.67	11.7
26^46^				Au/P3HT/perov/c‐TiO_2_/FTO	11.5	1.00	0.67	7.7
27^47^	CsPbIBr_2_		Spray‐coating	Au/spiro‐OMeTAD/perov/mp‐,c‐TiO_2_/FTO	7.0	1.08	0.72	5.4
28^48^		+SnBr_2_	Spin‐coating	Carbon/perov/mp‐,c‐TiO_2_/FTO	14.3	1.26	0.63	11.3

Unfortunately, the research level on CLH perovskites still lags behind that on organic perovskites. Main reason for this inferiority was a lack of efficiency and stability. Even though CsPbI_3_ possesses a suitable bandgap for solar cells (1.73 eV), it rapidly degrades to the non‐perovskite yellow phase (δ‐phase) in the ambient atmosphere. It is almost impossible to fabricate and operate CsPbI_3_‐based solar cells in a general experimental method. As discussed above, CsPbBr_3_ is highly stable material against air and moisture. However, the bandgap of this material (2.25 eV) limits the range of light absorbed. A study on cesium lead mixed‐halide perovskites provided a facile approach for balancing the bandgap (optical absorption) and structural stability. As a result, the relevant research on these materials has scarcely been reported.

**Figure 2:**
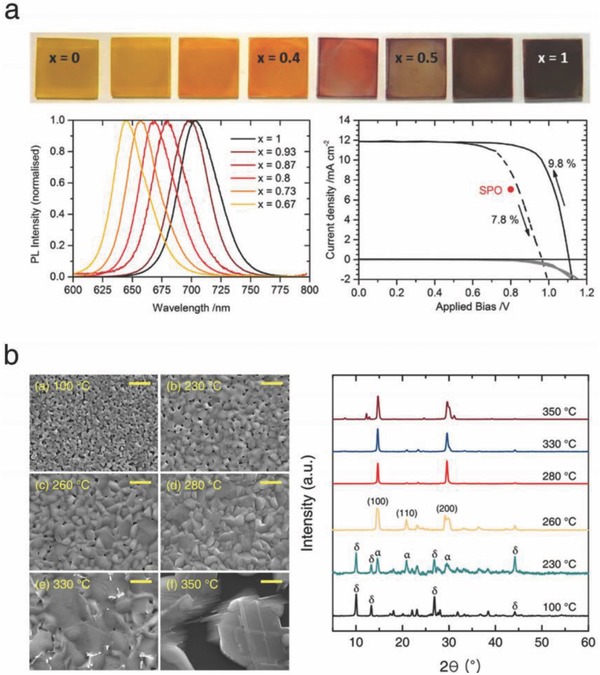
a) PL spectra of the CsPbI_3−_ *_x_*Br*_x_* films, and *J–V* scan of the CsPbI_2_Br‐based solar cell. Reproduced with permission.^21^ Copyright 2016, Wiley‐VCH. b) SEM images and XRD spectra of the differently annealed CsPbI_2_Br films. Reproduced with permission.^24^ Copyright 2017, American Chemical Society.

In early 2016, Sutton et al. studied the optoelectronic and structural properties of CsPbI_3−_ *_x_*Br*_x_*, and demonstrated the CsPbI_2_Br‐based solar cells.^21^ The photographs and photoluminescence (PL) spectra of the CsPbI_3−_ *_x_*Br*_x_* films confirmed that the optical absorption could be appropriately tuned for a specific purpose by varying the ratio of I and Br (**Figure** **2**a, left). In addition, the incorporation of Br into CsPbI_3_ stabilized the α‐phase even in moisture‐containing air. The tolerance factor, which is a determinant of structural stability, can be adjusted by the partial incorporation of Br, indicating that Cs cations favorably hold the contracted Pb(I_1−_ *_x_*Br*_x_*)_6_ (compared to bare CsPbI_3_) octahedra network. They also revealed that the phase transition temperature from the as‐formed δ‐phase to the α‐phase depends on the I/Br composition. Optimally, CsPbI_2_Br possesses a bandgap of 1.92 eV, which is appropriate for the top cell of silicon‐perovskite tandem devices, along with higher ambient stability. The solar cells based on this material exhibited a champion PCE of 9.8%, which was the highest record at that time (Figure 2a, right). At a similar time, same experimental result was reported by Beal et al.^22^

Our group further investigated the effect of annealing temperature on the crystal formation of CLH perovskites by observing the surface morphology, crystal structure, and chemical state of the differently annealed CsPbI_2_Br films (Figure 2b).^24^ At the optimal annealing temperature, the high‐quality film with pinhole‐free surface morphology and well‐oriented crystal structure was formed. It was verified that a moderate annealing temperature significantly reduced the surface and bulk defect sites (metallic Pb and halide vacancy) and preserved the cationic charge of Pb cations, thereby enhancing the photovoltaic performance as well as the phase stability. The optimized solar cell exhibited a PCE of 10.7%, and in particular, the *V*_OC_ of this device was 1.23 eV, which far outperformed that of the earlier reported devices.^21^, ^22^, ^23^ Overall, thanks to the high structural stability and moderate optical absorption of CsPbI_2_Br, the emergence of this material has led to remarkable efficiency records above 10% and accelerated subsequent studies in the field of all‐inorganic perovskite‐based photovoltaics.

**Figure 3:**
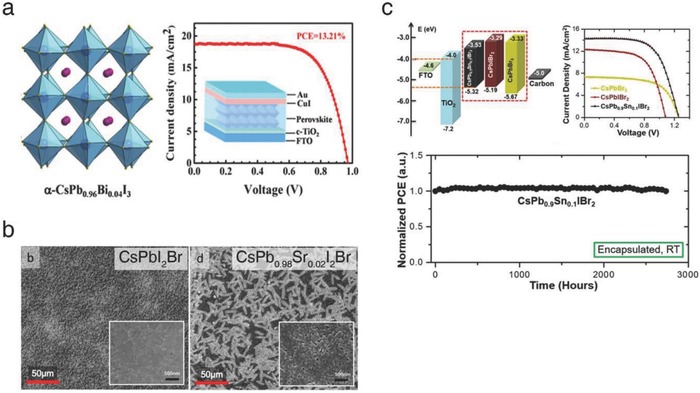
a) Distorted cubic structure of the α‐phase CsPb_0.96_Bi_0.04_I_3_ and corresponding *J–V* scan of the solar cell. Reproduced with permission.^30^ Copyright 2017, American Chemical Society. b) SEM images of the CsPbI_2_Br and CsPb_0.98_Sr_0.02_I_2_Br films. Reproduced with permission.^43^ Copyright 2017, American Chemical Society. c) Energy diagram and *J–V* scan of the CsPbBr_3_‐, CsPbIBr_2_‐, and CsPb_0.9_Sn_0.1_IBr_2_‐based solar cells. Normalized PCE of the encapsulated CsPb_0.9_Sn_0.1_IBr_2_‐based solar cell. Reproduced with permission.^48^ Copyright 2017, American Chemical Society.

The partial substitution of Pb in the B‐site of ABX_3_ perovskite lattice has been researched by several groups. First, Hu et al. reported Bi‐incorporated CsPbI_3_, investigating the crystal structure of CsPb_1−_ *_x_*Bi*_x_*I_3_ films.^30^ As the Bi mol% increased, CsPbI_3_ was gradually stabilized, showing the characteristic α‐phase peaks of (100), (110), (110), and (200) in the X‐ray diffraction (XRD) spectra. On the basis of this finding, a possible mechanism was proposed (**Figure** **3**a, left). Both intercalated HI and Bi induce the formation of microstrain within the perovskite lattice, facilitating the phase transition from the δ‐phase to α‐phase. In particular, a specific amount of Bi (4 mol%) leads to the distortion of CsPbI_3_ cubic lattice, resulting in further stabilized structure. The optimized CsPb_0.96_Bi_0.04_I_3_‐based solar cells exhibited a champion PCE of 13.2%, which is a record efficiency among all‐inorganic perovskite‐based solar cells reported so far (Figure 3a, right). In addition, the device showed improved ambient stability, maintaining 68% of its initial PCE for 168 h under 25 °C and 55% RH without encapsulation.

In addition, Lau et al. introduced Sr into CsPbI_2_Br.^43^ Low temperature‐processed CsPb_1−_ *_x_*Sr*_x_*I_2_Br films were investigated by examining the surface morphology, time‐resolved PL, and photovoltaic performance. Scanning electron microscopy (SEM) images of the Sr‐doped CsPbI_3_ films showed a distinctive morphology (snowflake shape) due to the enrichment of Sr (SrO) on the surface (Figure 3b). The PL decay profiles revealed that the surface passivation provided by SrO diminished defect‐driven non‐radiative recombination and increased effective charge carrier lifetime. At optimum, an average PCE of the solar cells were dramatically increased from 6.6 (CsPbI_2_Br) to 10.1% (CsPb_0.98_Sr_0.02_I_2_Br), including a champion of 11.3%. In addition, the encapsulated device remained its initial performance for 3 weeks (25 °C, <50% RH).

To tune the optical absorption and structural stability, Liang et al. studied the partial substitution of Sn in Br‐rich CLH perovskites.^48^ The incorporation of Sn into CLH perovskites effectively reduced the bandgap, similar to the mixed‐halide approach discussed above. Optimally, the bandgap of CsPb_0.9_Sn_0.1_IBr_2_ was measured to be 1.79 eV, close to that of CsPbI_3_ (1.73 eV) (Figure 3c, left). The film simultaneously exhibited superior stability thanks to the high Br content. Without a hole transport layer, the solar cells based on the CsPb_0.9_Sn_0.1_IBr_2_/carbon system exhibited a maximum PCE of 11.3% and a *V*_OC_ of 1.26 V, which was an exceptional record compared to the preceding literature (Figure 3c, right). In addition, the device showed an extremely long operational lifetime in the presence of heat and moisture (100 °C for 2 weeks and 60% RH for 50 h). In particular, the encapsulated device showed no degradation at room temperature and maintained its initial performance for more than 3 months (Figure 3c, bottom).

Besides, many other metal cations have been used to improve the quality of CLH perovskite films. In the early stage, our group reported K‐incorporated CsPbI_2_Br, improving the structural stability and charge transport.^23^ Recent studies showed that the partial replacement of Pb with Mn presented multiple benefits, stabilizing the α‐phase perovskite lattice, controlling the crystal growth, and tuning the optical absorption (bandgap).^44^, ^50^, ^51^ Similar improvements were achieved by incorporating Ca, achieving more than 13% efficient CsPbI_3_‐based solar cells.^31^

**Figure 4:**
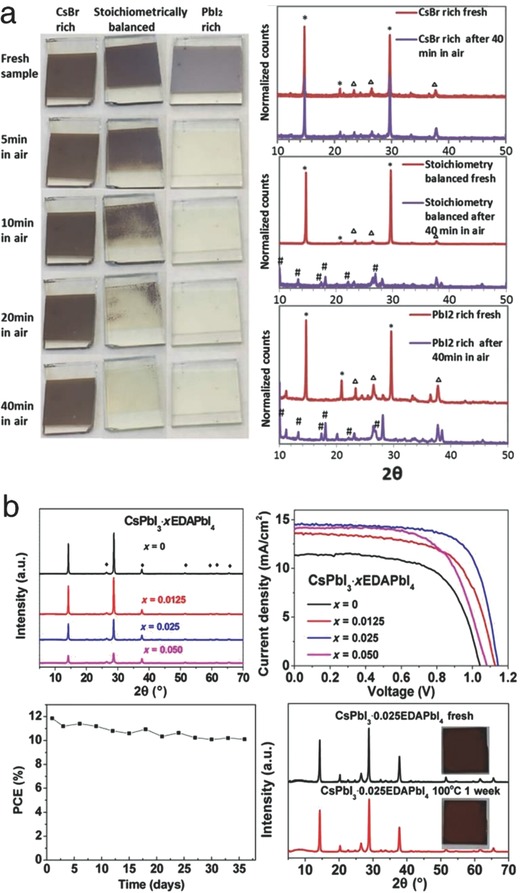
a) Photographs and XRD spectra of the stoichiometrically modified CsPbI_2_Br films (balanced, CsBr‐rich, and PbI_2_‐rich). Reproduced with permission.^46^ Copyright 2017, American Chemical Society. b) XRD spectra, *J–V* scans, and normalized PCE of CsPbI_3_∙xEDAPbI_4_‐based films and solar cells. Reproduced with permission.^33^ Copyright 2017, AAAS.

In another approach, the nonstoichiometric approach was performed by Ma et al., comparatively investigating the effect of varying the stoichiometry of CLH perovskite films.^46^ The stoichiometrically balanced, CsBr‐rich, and PbI_2_‐rich CsPbI_2_Br films were prepared by dual source thermal evaporation of precursors including CsBr and PbI_2_. The CsBr‐rich sample showed the best air stability among the samples, possibly due to its smaller grain size, which retarded structural degradation by inducing crystal lattice strain (**Figure** **4**a). In terms of efficiency, however, the stoichiometrically balanced sample, which was less stable, showed the best efficiency. The presence of impurities in the Cs‐rich and PbI_2_‐rich samples reduced the intensity and lifetime of PL, ultimately lowering the photovoltaic performance. This work revealed the relationship between the stoichiometry, photovoltaic performance, and structural stability through the integrated researches of crystallographic and photophysical properties.

In addition, Zhang et al. designed the 2D‐perovskite incorporated CsPbI_3_.^33^ A small amount of ethylenediamine (EDA) cations facilitated to form highly stabilized α‐phase CsPbI_3_ under the ambient atmosphere. The two terminal NH^3+^ groups of bication EDA occupied two A sites, resulting in the formation of A_2_BX_4_ 2D‐perovskites. The intercalated EDA cations in CsPbI_3_ diminished the undesirable phase transition to the δ‐phase by forming cross‐linked CsPbI_3_∙xEDAPbI_4_. The absence of any indexable impurity peaks in the XRD spectra verified that the added EDA cations were successfully incorporated in the perovskite lattice (Figure 4b, upper left). It was hypothesized that the layered EDAPbI_4_ might function as a passivation layer and significantly reduces the grain size. The solar cells based on optimized composition (*x* = 0.025) achieved a champion PCE of 11.8%, whereas the pure CsPbI_3_‐based cells exhibited a PCE of 7.7% PCE (Figure 4b, upper right). Furthermore, the device showed high stability against air (stored in a dry box for 1 month without encapsulation) and heat (100 °C for 1 week) (Figure 4b, bottom). Encouraged by this study, the combination of 2D‐ and 3D‐perovskites should be researched further to identify the structural evolution and its effectiveness on the structural stability and photovoltaic performance.

**Figure 5:**
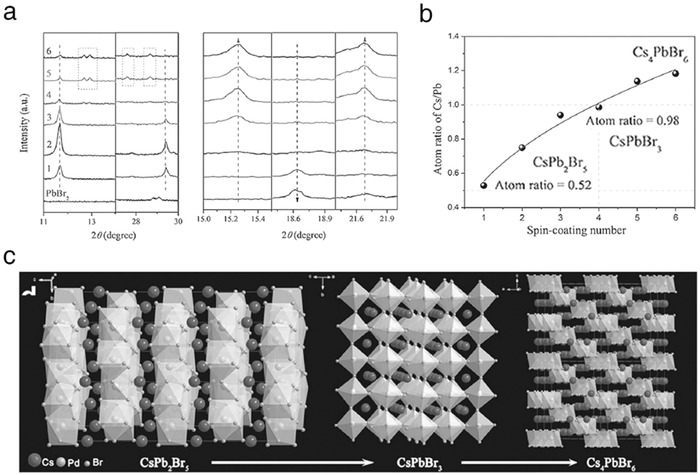
a) XRD spectra, b) atomic Cs/Pb ratios, and crystal structure of the Cs‐Pb‐Br perovskite films at different deposition cycles (*n* = 1 to 6). Reproduced with permission.^40^ Copyright 2018, Wiley‐VCH.

In another important aspect, the derivatives of CsPbBr_3_, including CsPb_2_Br_5_ and Cs_4_PbBr_6_, were studied.^40^, ^42^, ^52^, ^53^ Representatively, Duan et al. developed a sequential method to form high‐phase‐purity CsPbBr_3_ films by tuning the number of CsBr spin‐coating (*n*) onto a PbBr_2_ substrate.^40^ The transformation mechanism, including phase fusion and separation, was identified (**Figure** **5**).

(1) $$2{\mathrm{PbBr}}_{2}+\mathrm{CsBr}\to {\mathrm{CsPb}}_{2}{\mathrm{Br}}_{5}\left(n\leq 3\right)$$

(2) $${\mathrm{CsPb}}_{2}{\mathrm{Br}}_{5}+\mathrm{CsBr}\to 2{\mathrm{CsPbBr}}_{3}(n=4)$$

(3) $${\mathrm{CsPbBr}}_{3}+3\mathrm{CsBr}\to {\mathrm{Cs}}_{4}{\mathrm{PbBr}}_{6}\left(n\geq 5\right)$$

At *n* = 4, the films showed high‐phase‐purity crystal structure well as large grained surface morphology. For the device fabrication, a CsPbBr_3_/carbon system was employed to fabricate all‐inorganic–based solar cells. Additionally, to reduce the energy barrier between the TiO_2_ and perovskite films, graphene QDs were introduced as an interfacial decoration, ultimately boosting solar cell efficiency up to 9.7%. The device also exhibited superior thermal and moisture stability (80 °C for 40 d and 90% RH for 130 d, respectively).

**Figure 6:**
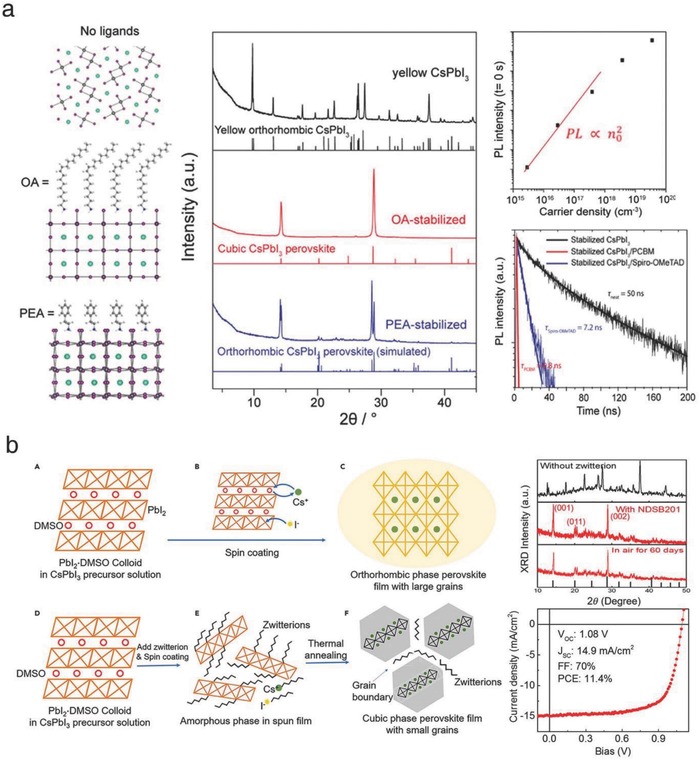
a) Schematic of the long‐chain ammonium‐driven stabilization of CsPbI_3_ and photophysical measurements of the PEA‐stabilized CsPbI_3_ films. Reproduced with permission.^32^ Copyright 2017, American Chemical Society. b) Hypothesized mechanism of zwitterion‐induced stabilization of CsPbI_3_. XRD spectra and *J–V* scan of the NDSB201‐incorporated CsPbI_3_ film and solar cell. Reproduced with permission.^34^ Copyright 2017, Elsevier.

Several additives have been used to control the crystal growth of perovskite films. First, Fu et al. introduced monovalent organic cations to stabilize the α‐phase of CLH perovskites. Long‐chain ammonium additives such as oleylammonium (OA) and phenylethylammonium (PEA) acetate were employed to form the stable CsPbI_3_ films.^32^ At a low annealing temperature (120 °C), the cubic α‐phase and orthorhombic β‐phase CsPbI_3_ films were formed, enabled by the addition of OA and PEA, respectively. Stability tests showed that both films remained their initial state for several months at room temperature. It was hypothesized that ammonium cations with extremely long chains could not intercalate into the perovskite lattice but, rather attached to the crystal surface as a capping ligand (**Figure** **6**a, left and middle). These kept halide perovskite films from further grain growth, ultimately stabilizing the phase structure. The photoexcited carrier dynamics of lower symmetry β‐CsPbI_3_ (PEA‐stabilized) was investigated, which was less commonly reported. Measurements of PL quenching and decay profiles verified that the additive‐mediated β‐CsPbI_3_ films exhibited efficient charge transport, possessing sufficient long‐lived charge carriers under solar illumination (Figure 6a, right). This study suggested that long ammonium cations may be novel additives for modulating the phase‐polymorph of CLH perovskites toward efficient and stable solar cells.

Similarly, Wang et al. added sulfobetaine zwitterions into the precursor solution to reduce the grain size of CLH perovskite films.^34^ The CsPbI_3_ films incorporating several zwitterions exhibited much smaller grains than the pristine one. In particular, 3‐(1‐pyridinio)‐1‐propanesulfonate (NDSB201) helped form the α‐phase of CsPbI_3_ at a relatively low annealing temperature (80 °C, vs 350 °C for pristine).^21^ The film also maintained its initial crystal structure for 60 d in the ambient atmosphere. It can be concluded that the additive‐driven crystal growth inhibition improves the phase stability. On the basis of this observation, it was hypothesized that zwitterions in the precursor interacted with the PbI_2_‐DMSO complex and broke its layered structure, effectively impeding the rapid grain growth within the spin‐coated CsPbI_3_ film. During the annealing step, the intercalated molecules were expected to be expelled to the grain boundaries and surfaces to maintain a small grain size and inhibit further growth (Figure 6b, left). In this regard, for device fabrication, a plasma treatment was introduced to partially remove residuals from the surface, which are detrimental to charge carrier extraction. The solar cell employing NDSB201‐incorporated CsPb(I_0.98_Cl_0.02_)_3_ exhibited a champion PCE of 11.4% and maintained 85% of its initial performance over 30 d (in the ambient atmosphere without encapsulation) (Figure 6b, right). The introduction of molecular ligands may be a facile means for identifying and controlling crystal formation in solution‐processed CLH perovskite films.

In a short time, the efficiency and stability of CLH perovskite‐based solar cells have dramatically improved as discussed in this section. The attempts to stabilize the α‐phase of pristine CsPbI_3_ and CsPbI_2_Br have facilitated the fabrication of ambient stable, over 10% efficient solar cells under moderate experimental condition. The partial substitution of cations or halides in CLH perovskites has simultaneously balanced the optical absorption (bandgap) and structural stability. In particular, recent reports have emphasized that the introduction of metal cations could lead to the distortion of the perovskite lattice, possibly improving the interaction between A‐site cations (Cs) and inorganic octahedra network (PbX_6_, X = I, Br). In addition, specific additives, including polymers, ammonium salts and ligands, have helped impede excessive crystal growth, resulting in further stabilization of the extremely unstable CsPbI_3_ films. The works presented here successfully fabricated ambient stable α‐phase CLH perovskite films at a low annealing temperature (100 °C), which is far lower than those employed for the previously reported ones (300 °C). Certainly, this is very advantageous in terms of device fabrication, utilizing polymer‐based flexible substrates and organic charge transport materials. The state‐of‐the‐art compositional engineering of CLH perovskites will lead to further progress, for example, by the introduction of alkali metals (Rb, K), substitution of Pb with other triple‐valent cations (Sn, Mn, Sb, Ga, Al, etc.), and addition of chemical additives, following the successful history of organic perovskites.^16^, ^17^, ^54^, ^55^, ^56^, ^57^

## 3 Deposition Method

In organic perovskite‐based solar cells, the state‐of‐the‐art deposition methods, such as two‐step sequential method, thermal evaporation, and antisolvent‐washing have been introduced to overcome the solubility difference between organic (MAI or FAI) and inorganic (PbI_2_) components.^4^, ^5^, ^6^, ^10^ Comparatively, in CLH perovskite‐based solar cells, one of the major constraints is the solubility limit of Cs and Br precursors. Former reports have stated that a lack of solubility hindered the fabrication of a sufficiently thick and pinhole‐free perovskite film.^21^, ^22^, ^23^, ^24^ Our previous study verified that at most 0.4 m CsPbI_2_Br precursor solution could be made, and it formed an only 85 nm thick film by a one‐step spin‐coating method.^24^ The introduction of a well‐modulated solvent system or vapor‐based vacuum deposition could be a practical solution to this issue. In this section, the recent achievements in solution‐, vacuum‐, and colloidal QD‐based methods for the fabrication of CLH perovskite films are summarized.

**Figure 7:**
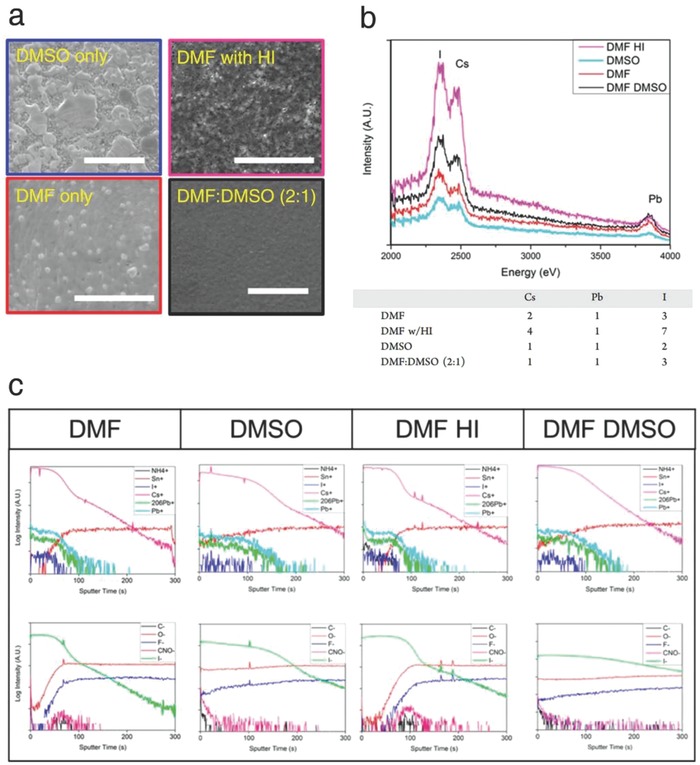
a) SEM images, b) LEIS intensities, and c) SIMS measurements of the CsPbI_3_ films employing different solvent systems. Reproduced with permission.^58^ Copyright 2017, American Chemical Society.

For the solution‐processed spin‐coating method, Ramadan et al. studied the solvent dependence of the electronic and structural properties of CLH perovskite films.^58^ By comparing a range of solvent systems such as dimethyl formamide (DMF), dimethyl sulfoxide (DMSO), and a mixture of two, substantial chemical and compositional differences in the CsPbI_3_ films were revealed. SEM images showed that the precursor with the DMF:DMSO mixed‐solvent (optimum volumetric ratio of 2:1) produced a high‐quality CsPbI_3_ film with full coverage, while employing DMF‐ or DMSO‐only the solvent resulted in an incomplete and rough surface with pinholes (**Figure** **7**a). Furthermore, low energy ion scattering (LEIS) and secondary ion mass spectrometry (SIMS) measurements identified that the DMF:DMSO mixed‐solvent yielded better CsPbI_3_ crystallinity. The LEIS intensity of Cs, Pb, and I ions gave the stoichiometric data, supporting that DMF:DMSO mixed‐solvent formed stoichiometrically balanced CsPbI_3_ (molar ratio of 1:1:3 for Cs:Pb:I) (Figure 7b). In addition, the depth profiling by time‐of‐flight SIMS exhibited a gradual slope for Cs, Pb, and I ions within the films, indicating an even distribution of the elements (Figure 7c). As suggested in this report, further understanding and modulating the solvent system for solution‐processed CLH perovskite films will contribute to significant improvements in the photovoltaic performance.

**Figure 8:**
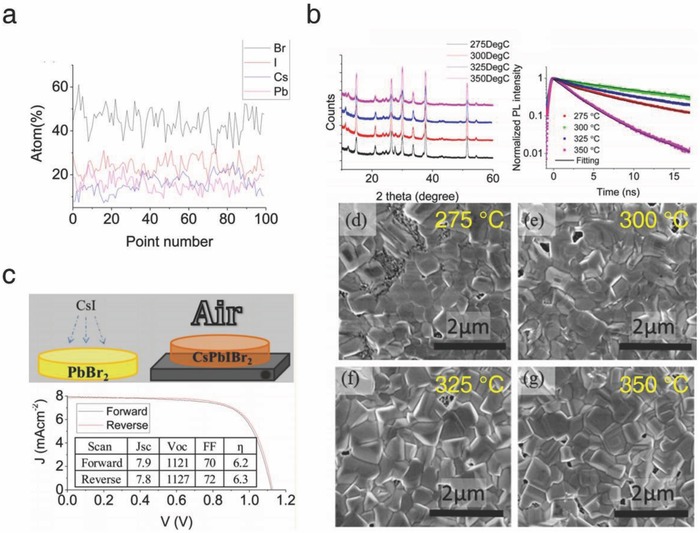
a) EDS spectra, b) XRD spectra and corresponding SEM images of the spray‐coated CsPbI_2_Br films. c) *J–V* scan of the optimized solar cell. Reproduced with permission.^47^ Copyright 2016, American Chemical Society.

To overcome the solubility limits mentioned above, Lau et al. utilized a combination of spin‐coating and spray deposition methods.^47^ The CsPbIBr_2_ film was fabricated by a two‐step process: First, the PbBr_2_ film was deposited on a TiO_2_ substrate by spin‐coating. Subsequently, the solution of CsI dissolved in methanol was sprayed onto the as‐formed film. Annealing was then performed in the range of 275 to 350 °C. Energy dispersive spectroscopy measurements verified that the ultimately fabricated film at room temperature showed well‐balanced stoichiometry (Pb/Cs = 1, Br/I = 2) (**Figure** **8**a). SEM images and PL decay profiles confirmed the optimum annealing temperature of 300 °C, which gave a pinhole‐free surface morphology and the longest charge carrier lifetime (Figure 8b). The fabricated solar cells exhibited a stabilized PCE of 6.3% with negligible hysteresis behavior (Figure 8c).

**Figure 9:**
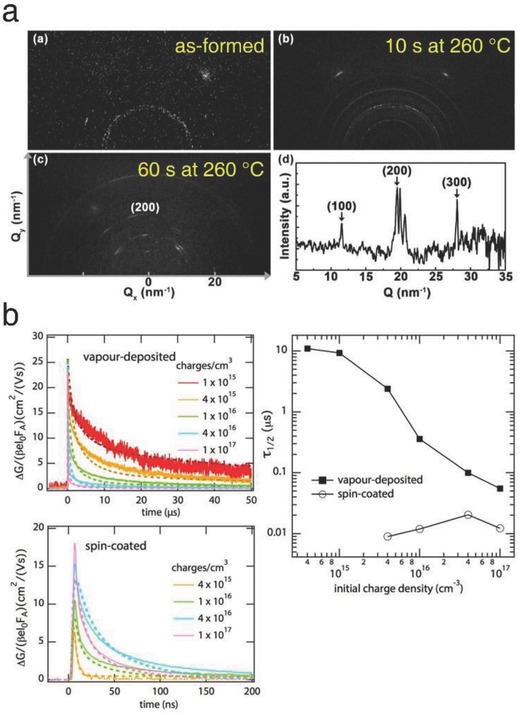
a) GIWAXS patterns of the vacuum‐deposited CsPbI_2_Br films. Reproduced with permission.^27^ Copyright 2017, Wiley‐VCH. b) TRMC curves of the vapor‐ and solution‐deposited CsPbI_3_ films. Reproduced with permission.^36^ Copyright 2017, American Chemical Society.

Vacuum‐processed vapor deposition was introduced to form a sufficiently thick and highly uniform perovskite film. First, Chen et al. designed a co‐sublimation deposition method for halide precursors (CsI, CsBr, PbI_2_, and PbBr_2_) in a custom‐made vacuum chamber.^27^ Ellipsometry data identified the correct calibration of CLH perovskite thin films. By integrating this technique with vacuum‐sublimed charge transport layer, all‐vacuum‐deposited and stoichiometrically balanced CLH perovskite‐based solar cells were successfully fabricated. The precise control of the co‐deposition of the precursors was identified to be essential for the formation of a film with the desired crystal structure. The solar cells employing the stoichiometrically balanced CsPbI_3_ exhibited the highest PCE of 9.4%, outweighing those of the unbalanced ones. Furthermore, the effect of annealing temperature on the vacuum‐deposited CsPbI_2_Br films was investigated by grazing‐incidence wide‐angle X‐ray scattering (GIWAXS) measurements. The optimally annealed film exhibited the preferred orientation and a well‐ordered cubic structure (scattering vector, Q = 20 nm^−1^ for (200)) (**Figure** **9**a). On the basis of this finding, the optimized solar cells achieved a champion PCE of 11.8% and a stabilized efficiency above 11% with negligible hysteresis, which was not achieved by solution processing. It is inferred that the period of air exposure during the deposition process can have a profound effect on the quality of CLH perovskite films, since halide precursors are chemically vulnerable to air and moisture.

Utilizing a similar system, Hutter et al. compared the charge carrier dynamics in the CsPbI_3_ thin films formed by vapor‐ and solution‐deposition routes.^36^ The time‐resolved microwave conductivity (TRMC) technique identified that the charge carrier mobility was approximately 25 cm^2^, for both the vapor‐ and solution‐deposited films (Figure 9b, left). However, an exceptionally long lifetime on the order of tens of microseconds was observed in the vapor‐deposited film, whereas for the solution‐deposited film, all the photoexcited carriers were immobilized within 200 ns. The fitted trap densities based on the TRMC curves were calculated to be 1:1 × 10^16^ and 9 × 10^14^ cm^−3^ for the vapor‐ and solution‐deposited films, respectively. It could be explained that a large portion of free charges were rapidly trapped by the defect sites formed in the low‐quality solution‐processed film. This assumption was further supported by the half lifetime curves measured as a function of the initial charge excitation density (Figure 9b, right). Higher‐order recombination kinetics was observed in the vapor‐deposited film, whereas the gradual filling and saturation of trap states were observed in the solution‐processed film, which verified the superiority of the vacuum‐based vapor deposition method.

**Figure 10:**
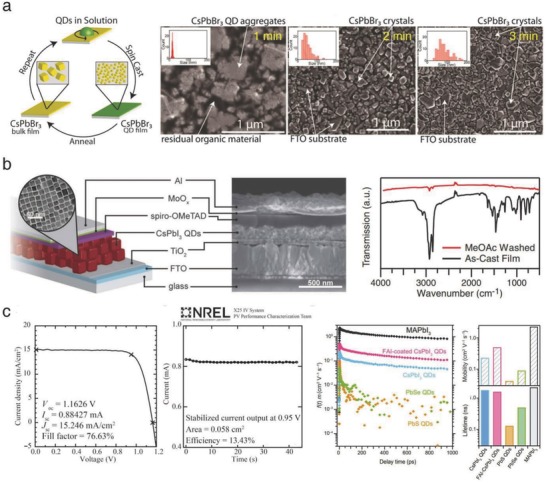
a) Schematic and SEM images of the layer‐by‐layer‐deposited CsPbBr_3_ QD films. Reproduced with permission.^25^ Copyright 2017, American Chemical Society. b) Device structure of the CsPbI_3_ QD‐based solar cell and FTIR spectra of the CsPbI_3_ QD films before and after dipping in MeOAc. Reproduced with permission.^26^ Copyright 2017, AAAS. c) *J–V* scan and stabilized efficiency of the CsPbI_3_ QD‐based solar cell and time‐resolved photoconductivity of the various QD‐based films. Reproduced with permission.^35^ Copyright 2017, AAAS.

Colloidal QD‐based thin film fabrication was introduced to fabricate ambient stable CLH perovskite‐based solar cells. Previous studies experimentally supported the fact that the size‐confined perovskite QDs exhibited a higher α‐phase stability than the bulk film, thanks to the large contribution of the surface energy.^20^, ^59^, ^60^ In addition, the well‐established synthetic technique is highly beneficial for modulating the size, bandgap, and surface properties of halide perovskite colloids. First, Hoffman et al. designed a layer‐by‐layer deposition process of repeated spin‐coating and annealing of CsPbBr_3_ QDs on a substrate (**Figure** **10**a, left).^25^ The transformation from the as‐deposited QDs to the bulk film was investigated through SEM surface images (Figure 10a, right). At an early stage of annealing, the CsPbBr_3_ QDs aggregated into clusters, while residual organic molecules still existed. After a short annealing period, the QD clusters were transformed into large crystals (average size of 85 nm) and the residuals were removed, forming a typical crystalline grain‐containing film. The solar cells fabricated through this method achieved a champion PCE of 5.6% and a *V*_OC_ of 1.42 V. They suggested that further optimization should be required to remove undesirable residuals and form smooth, pinhole‐free and highly crystalline CLH perovskite films.

Compared to Br^−^ in CsPbBr_3_, I^−^ in CsPbI_3_ forms weaker acid‐base interactions with oleylammonium molecules, which is a typical surface ligand of CLH perovskite QDs. As a result, the synthesis of fully isolated CsPbI_3_ QDs is highly challenging since agglomeration occurs easily and causes a rapid phase transition from the α‐phase to the δ‐phase. To overcome this challenge, Swarnkar et al. introduced methyl acetate (MeOAc) as a washing agent to fabricate phase‐stable CsPbI_3_ QD films and examined their solar cell application.^26^ In general, the removal of unreacted precursors and organic residuals requires an annealing procedure above 200 °C; however, this procedure causes further grain growth, ultimately leading to phase degradation. Instead, dipping in MeOAc effectively eliminated the organic ligands in the as‐deposited films, helping form highly phase‐stable CsPbI_3_ QDs at a low annealing temperature (Figure 10b, left). It was also revealed that a saturated solution of Pb^2+^ salts in MeOAc exhibited vastly improved surface passivation compared to that of the pristine solution, resulting in a PL enhancement. On the basis of their development, the fabricated solar cells achieved a champion PCE of 10.8% and maintained the initial efficiency more than 2 weeks (Figure 10b, right).

Recently, in work by the same group, the CsPbI_3_ QD‐based solar cells were further improved by AX post‐treatment (A = MA, FA, or Cs and X = I or Br), facilitating electronic interaction between QDs.^35^ After fabricating a QD film on a substrate, the film was immersed in an AX salt solution for a short time. Among the candidates, the solar cells employing FAI‐treated CsPbI_3_ QD films achieved a certified PCE of 13.4%, which was the highest value achieved among the reported colloidal QD‐based solar cells at that time (Figure 10c, left). Time‐resolved terahertz spectroscopy (TRTS) was performed to compare the charge transport behavior of several QD‐based films. It was identified that the mobility of the pristine CsPbI_3_ QD film was much higher than that of other typical Pb‐based chalcogenide QD films (0.23 cm^2^ V^−1^ s^−1^ for CsPbI_3_, vs 0.042 and 0.090 cm^2^ V^−1^ s^−1^ for PbS and PbSe, respectively) (Figure 10c, right).^61^ The mobility was further enhanced by the FAI treatment, reaching 0.50 cm^2^ V^−1^ s^−1^ for CsPbI_3_. A better understanding of charge transport between QD‐QD interfaces might be a key to the fabrication of highly efficient QD‐based solar cells. In this regard, this study provides a facile but effective strategy for enhancing the carrier mobility between QDs without a loss in the phase stability.

Each of the methods presented here can facilitate not only experimental‐level, but also industrial‐level fabrication of CLH perovskite‐based solar cells. First, the solution process, especially spray‐assisted coating, is a facile route to scalable fabrication. The precise control of the experimental atmosphere, including humidity and temperature, is required to reproducibly fabricate high‐quality thin films. In the case of vacuum deposition, there can be many limitations, such as the cost of the facility and the difficulty in large‐area fabrication. It is also difficult to control the stoichiometry of each precursor. Instead, this method is highly beneficial for utilizing the well‐established industrial production technology. Finally, the colloidal QD‐based thin films exhibit superior stability thanks to their size and surface effect. The synthetic technique and surface chemistry will improve the optoelectronic performance of CLH perovskite QD‐based solar cells. However, the scalable production of QDs and novel deposition method for fabricating large‐area and densely packed films must be developed. Undoubtedly, the development and optimization of fabrication processes is an essential step for achieving highly efficient and scalable CLH perovskite based solar cells. Due to a lack of research effort, a huge deviation in the experimental details still remains.^21^, ^24^, ^27^ However, impressive achievements have already been made and this system will be improved further.

## 4 Photophysical Analysis

The relationship between the crystal characteristics and charge carrier kinetics is a key reason for the superiority of halide perovskites.^61^ Early works on the photophysics of halide perovskites suggested that the organic cation (MA or FA)‐induced molecular dipole should be an intrinsic factor, and several theoretical and experimental studies supported this assumption.^62^, ^63^, ^64^, ^65^, ^66^, ^67^ This premise is still controversial, however, as the recent observations on photoinduced charge carrier kinetics have reported that nondipolar cesium‐based perovskites also exhibit possess exceptionally slow charge carrier recombination and facile transport. For instance, Hutter et al. suggested that the charge carrier kinetics should be determined by an inorganic octahedra network (PbX_6_, X = I, Br), not by the A‐site monovalent cation (MA, FA, or Cs).^36^, ^68^ In addition, several studies have discovered that the ion migration behavior is quite different between CLH perovskites and the organic counterparts, which is importantly featured in this section.

**Figure 11:**
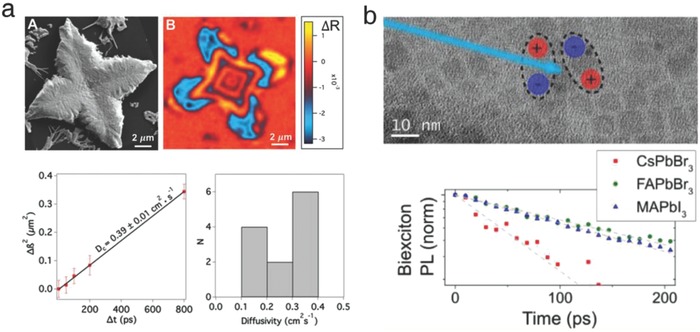
a) SEM image, TR response, and diffusivity of the CsPbI_2_Br single domain. Reproduced with permission.^69^ Copyright 2017, American Chemical Society. b) Schematic of biexcitons and PL decay profiles of various halide perovskite QDs. Reproduced with permission.^76^ Copyright 2017, American Chemical Society.

First, the charge carrier recombination kinetics of CLH perovskites was investigated by pump‐probe microscopy. Kennedy et al. studied the ultrafast excited‐state dynamics by spatially probing mixed‐halide CLH perovskites.^69^ An isolated and microsized CsPbI_2_Br single crystal, prepared by spin‐coating, was characterized by capturing a transient reflectivity (TR) image (**Figure** **11**a, upper). In general, the TR response reflects the photophysical differences between the equilibrium and excited state within the semiconductors, as modeled by the following expression

(4) $$\frac{\partial N\left[t\right]}{\partial t}=-k_{1}\cdot N\left[t\right]-k_{2}\cdot N{\left[t\right]}^{2}-k_{3}\cdot N{\left[t\right]}^{3}$$

where *N*[t] is the photoexcited carrier density at a given time, *t*, and *k*_1_, *k*_2_, and *k*_3_ represent the first‐, second‐, and third‐order recombination rate constants, respectively.^63^, ^70^, ^71^ The fitted parameters based on the power‐dependent kinetic model (Equation (1)) implied that the bimolecular (second‐order) mechanism, representing a direct recombination across the bandgap, was negligible. Instead, the overall charge carrier lifetime was dominated by the Shockley–Read–Hall (SRH, first‐order) and Auger (third‐order) mechanisms, representing a trap‐mediated nonradiative recombination, as identified by the time‐correlated single photon counting data. However, according to the previous reports, the α‐phase tetragonal MAPbI_3_ showed extremely slow recombination, inhibiting the trap‐mediated route.^71^, ^72^, ^73^, ^74^ It was also noteworthy that the decay of MAPbI_3_ at low temperature (orthorhombic phase, exhibiting smaller dielectric constants as a result of an ordered MA distribution) followed similar kinetics as those observed in CsPbI_2_Br.^75^ In addition, the diffusion constant of CsPbI_2_Br was calculated to be 0.39 cm^2^ s^−1^, which was significantly lower than that of MAPbI_3_ (1.77 cm^2^ s^−1^, measured by the same group) (Figure 11a, bottom).^67^ Although the influence of the halide composition could not be ignored, the composition‐dependent molecular interactions might play a substantial role in determining the charge carrier behavior of halide perovskites.^66^

Recently, Eperon et al. compared Auger recombination in organic and CLH perovskite QDs.^76^ By integrating the time‐resolved PL data, it was identified that the organic perovskites, containing polar molecules (MAPbBr_3_ and FAPbBr_3_), showed much smaller Auger rates (longer lifetime) than the all‐inorganic analogues (CsPbBr_3_, rapid decay) (Figure 11b). Furthermore, the volume dependence was far weaker in organic perovskites, suggesting that the organic cations might screen and mediate charge carrier interactions.^66^, ^67^ The liquid‐like molecular reorientation promoted the formation of solvated ions or large polarons, effectively protecting the photoexcited electrons and holes from recombination. It was also inferred that cesium‐based perovskites should possess a higher defect density since they exhibited larger volume dependence. It was noted that this study provided a photophysical observation regarding the role of organic cations; however, the fundamental difference between the organic and inorganic perovskites was not resolved and therefore deserves further research.

**Figure 12:**
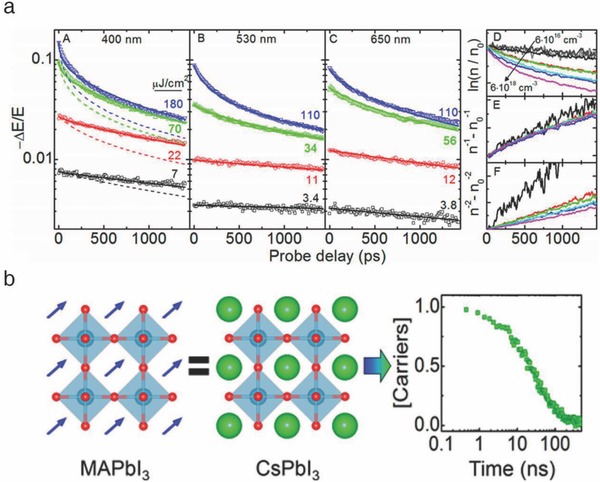
a) TRTS curves for the different pump pulses of the CsPbI_3_ film. b) Molecular structure of MAPbI_3_ and CsPbI_3_, and transient absorption decay of the CsPbI_3_ film under 650 nm excitation. Reproduced with permission.^77^ Copyright 2017, American Chemical Society.

In contrast, Dastidar et al. suggested that CLH perovskites should exhibit charge carrier kinetics similar to those of their organic counterparts.^77^ The recombination kinetics were studied by doping different initial photoexcited carrier concentrations on a solution‐processed CsPbI_3_ film (**Figure** **12**a). Analysis of the TRTS signal confirmed that the bimolecular rate constants, *k*_2_, (modeled similarly to Equation (1)) was identical for CsPbI_3_ and MAPbI_3_, and exceptionally small comparable to that of single‐crystalline GaAs. Moreover, highly sample‐dependent behavior was observed in the monomolecular rate constant, *k*_1_, showing a comparable value to that of MAPbI_3_ on occasion. The optimized film exhibited a half‐life longer than 20 ns, supporting the premise that there is no fundamental difference in the photophysical behaviors between halide perovskites with and without a molecular dipole (Figure 12b). It has already been shown by the early studies of organic perovskites that, this kind of defect site could be significantly reduced by developing and optimizing the fabrication process.

**Figure 13:**
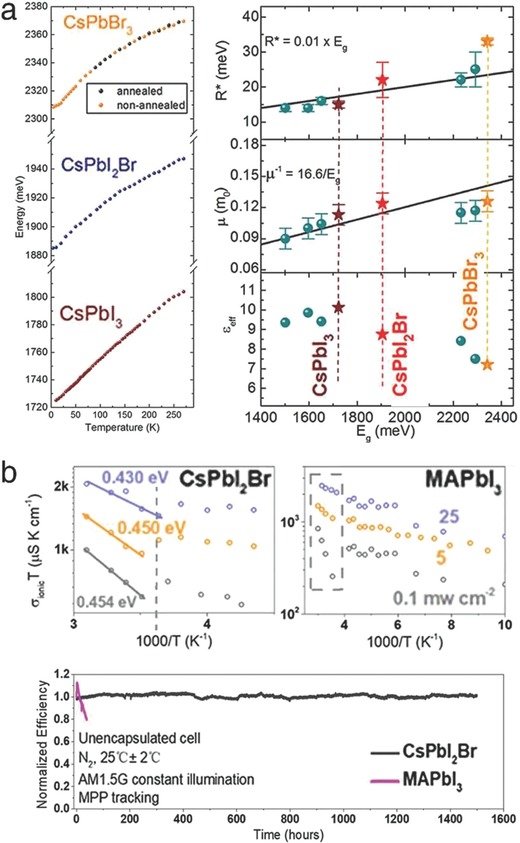
a) Transmission spectra over a wide temperature range (4.2‐280 K) and calculated electronic parameters: exciton binding energy (R*), effective mass [μ(*m*_0_)], and dielectric constants (ε_eff_) of the CsPbI_3_, CsPbI_2_Br, and CsPbBr_3_ films. Reproduced with permission.^78^ Copyright 2017, American Chemical Society. b) Measurements of the ionic conductivity and normalized efficiency of the CsPbI_2_Br‐ and MAPbI_3_‐based films and solar cells. Reproduced with permission.^28^ Copyright 2017, American Chemical Society.

More fundamentally, Yang et al. performed the magneto‐optical investigation to determine the electronic properties of CLH perovskites.^78^ The mixed‐halide CsPbX_3_ films (CsPbI_3_, CsPbI_2_Br, and CsPbBr_3_) were prepared to compare the dependence of the photophysical properties on the halide cage (octahedra network of PbX_6_, X = I, Br). The transmission spectra did not show any structural phase transitions even at extremely low temperature, in contrast to those of the organic counterparts as previously reported (**Figure** **13**a, left).^14^, ^79^, ^80^ It was suggested that the presence of a molecular dipole determines the overall electronic properties of halide perovskites. Next, the major electronic parameters were measured, and they exhibited a well‐matched linear dependence on the temperature (Figure 13a, right). Integrating this result with a comparable study on organic perovskites, it was identified that the CLH perovskites maintained the α‐phase (cubic for CsPbI_3_ and orthorhombic for CsPbBr_3_) at low temperature, whereas the organic analogues underwent phase transition to a lower symmetry structure (tetragonal to orthorhombic).^79^ Furthermore, the dielectric constants, ε_eff_, exhibited a significant halide dependence. Comparing the CLH perovskites with their organic counterparts, this value was fairly similar for the iodide‐rich compounds but dramatically decreased for the bromide‐rich ones. Overall, this study identified that in the absence of molecular motion, the electronic interactions in the halide perovskite lattice are determined primarily by the halide composition, not by the A‐site monovalent cation. Additionally, the comparably small exciton binding energy (R*) in CsPbI_3_ showed its capability for functioning as highly efficient solar cell materials.

Finally, a comparison of the ion migration behavior between organic‐ and CLH perovskites was quantitatively performed by Zhou et al., by calculating the ionic conductivity as a function of temperature and light intensity.^28^ The activation energy for ion migration (*E*_a_) could be derived from the equation

(5) $$\sigma_{\mathrm{ion}}\left(T\right)=\sigma_{0}\exp\left(\frac{E_{a}^{\mathrm{eff}}}{k_{B}T}\right)$$

The ionic conductivity, σ_ion_, was obtained by separating the electron conductivity through galvanostatic measurement (σ_ion_ = σ_total –_σ_electron_). The fitted parameters verified that the energy barrier for ion migration in MAPbI_3_ decreased from 0.62 to 0.07 eV after light illumination, whereas that in CsPbI2Br remained constant (around 0.45 eV) (Figure 13b, upper). This result could be interpreted as the fact that light illumination significantly activated the ion migration in MAPbI_3_ but not in CsPbI_2_Br. This difference was further supported by the PL measurements, exhibiting suppressed photoinduced halide segregation in the CLH perovskites. The stability of the solar cells was examined under continuous 1 sun illumination, showing that the CsPbI_2_Br‐based solar cell retained its initial PCE for 1500 h, while the MAPbI_3_‐based solar cell severely degraded within 50 h (Figure 13b, bottom). It was somewhat encouraging that CLH perovskites could be a potential alternative to organic perovskites as photostable solar cell materials.^81^, ^82^ The soft nature of organic molecules and dipole‐induced reorientation might be responsible for this behavior, which is supported by the preceding literature.^66^ In fact, further investigation should be performed to identify the origin of the distinguishable photophysics in organic‐ and CLH perovskites.

## 5 Conclusion and Perspectives

This review has discussed the recent progress in CLH perovskite‐based solar cells. The related studies are categorized into four sections: First, the compositional modification of CLH perovskites has been studied. In the early stage, the mixed‐halide approach showed a numerous potential for developing CLH perovskites as stable and efficient solar cell materials by successfully fabricating ambient stable, and over 10% efficient solar cells.^21^, ^22^, ^23^, ^24^ This advance encourages follow‐up studies in the field of all‐inorganic perovskite‐based solar cells. The partial substitution of Pb in the B‐site of ABX_3_ perovskites was shown to have a significant effect on both the photovoltaic performance and structural stability. In particular, Sn‐incorporated mixed‐halide CLH perovskites (CsPb_0.9_Sn_0.1_IBr_2_) exhibited a well‐balanced bandgap and structural stability.^48^ In addition, several strategies for stabilizing the α‐phase of CsPbI_3_ by chemical additives have been introduced. Intercalated molecules, such as 2D‐perovskites, ammonium salts and zwitterions led to the formation of stable α‐phase CsPbI_3_ at a low annealing temperature of approximately 100 °C in the ambient atmosphere.^32^, ^33^, ^34^, ^55^ Second, the solution‐ and vacuum‐processed deposition methods have been covered. The systematic studies on the solvent system have provided a guideline for improving the quality of solution‐processed CLH perovskite films. The spray‐coating method has successfully been demonstrated, overcoming the solubility limit of precursors as well as providing a possibility for the large‐area fabrication.^47^ Moreover, the application of thermal co‐evaporation enables the deposition of highly crystalline and stoichiometry‐controlled CLH perovskite films and is likely to be adequate for industrial production.^27^, ^36^, ^37^ In addition, the QD‐based fabrication provides new ways of introducing colloidal chemistry into photovoltaics.^25^, ^26^, ^35^ Finally, the photophysical properties of CLH perovskites have been featured. Several studies have verified that CLH perovskites exhibit substantial optoelectronic properties including charge carrier recombination and transport behaviors comparable to those of organic perovskites.^28^, ^59^, ^66^, ^69^, ^76^, ^77^, ^78^ Consequently, CLH perovskites has shown better photostability thanks to their insensitivity to photoinduced ion migration. All these achievements and observations have been made in a very short period, thereby illustrating the potential for further improvements and for ultimately catching up with the photovoltaic performance of organic perovskites.

No fundamental differences in the charge carrier kinetics between organic and CLH perovskites implies that the molecular dipole present in the organic cations may not be vital for the superiority of perovskite‐based solar cells. Rather, CLH perovskites have a crucial benefit of photostability due to the inhibition of ion migration, whereas in organic perovskites, ion migration is the major cause of photoinduced degradation. In terms of the conversion efficiency, the large bandgap of CLH perovskites may not be ideal for a single solar cell. For example, the Schottky‐Queisser limit implies that a bandgap of 1.92 eV (CsPbI_2_Br) should produce a theoretical maximum *J*_SC_ and *V*_OC_ of 16.3 mA cm^−2^ and 1.63 V, respectively, which implies that CsPbI_2_Br may not be able to compete with the efficiency of organic perovskites.^21^, ^23^, ^24^ Instead, the high *V*_OC_ of the device is beneficial for tandem applications, such as multijunction perovskite/silicon solar cells and integrated devices with photo‐electrochemical energy conversion cells. Considering the comparable efficiency with a large bandgap, this material also has advantages of dim‐light or indoor energy harvesting, inspiring next‐generation devices, particularly, self‐powered mobile electronics.

The structural instability under the ambient atmosphere and difficulty in fabrication due to the extremely high phase transition temperature has consistently been solved by compositional modifications and deposition techniques. The fabrication methods including solution‐, vacuum‐, and colloidal‐based processes have been successfully demonstrated. However„ the photophysical relationship between organic and all‐inorganic metal‐based perovskites should be further identified to reveal the cation‐dependent characteristics, thereby facilitating improvements in CLH perovskite‐based solar cells. In particular, along with their superior thermal stability, their photostable features, as revealed by the recent reports, will provide new possibilities for commercializing halide perovskite‐based photovoltaic devices.

## Conflict of Interest

The authors declare no conflict of interest.
